# The effect of temperature conditioning (9°C and 20°C) on the proteome of entomopathogenic nematode infective juveniles

**DOI:** 10.1371/journal.pone.0266164

**Published:** 2022-04-07

**Authors:** Peter E. Lillis, Christine T. Griffin, James C. Carolan

**Affiliations:** Department of Biology, Maynooth University, Maynooth, County Kildare, Ireland; University of Massachusetts Medical School, UNITED STATES

## Abstract

Entomopathogenic nematodes (EPN) of the genera *Steinernema* and *Heterorhabditis* are parasites which kill and reproduce within insects. While both have life cycles centred around their developmentally arrested, nonfeeding and stress tolerant infective juvenile (IJ) stage, they are relatively distantly related. These IJs are promising biocontrol agents, and their shelf life and stress tolerance may be enhanced by storage at low temperatures. The purpose of this study was to investigate how the proteome of the IJs of two distantly related EPN species is affected by storage at 9°C (for up to 9 weeks) and 20°C (for up to 6 weeks), using label-free quantitative proteomics. Overall, more proteins were detected in *S*. *carpocapsae* (2422) than in *H*. *megidis* (1582). The *S*. *carpocapsae* proteome was strongly affected by temperature, while the *H*. *megidis* proteome was affected by both time and temperature. The proteins which increased in abundance to the greatest extent in *S*. *carpocapsae* IJs after conditioning at 9°C were chaperone proteins, and proteins related to stress. The proteins which increased in abundance the most after storage at 20°C were proteins related to the cytoskeleton, cell signalling, proteases and their inhibitors, which may have roles in infection. The proteins which decreased in abundance to the greatest extent in *S*. *carpocapsae* after both 9°C and 20°C storage were those associated with metabolism, stress and the cytoskeleton. After storage at both temperatures, the proteins increased to the greatest extent in *H*. *megidis* IJs were those associated with the cytoskeleton, cell signalling and carbon metabolism, and the proteins decreased in abundance to the greatest extent were heat shock and ribosomal proteins, and those associated with metabolism. As the longest-lived stage of the EPN life cycle, IJs may be affected by proteostatic stress, caused by the accumulation of misfolded proteins and toxic aggregates. The substantial increase of chaperone proteins in *S*. *carpocapsae*, and to a greater extent at 9°C, and the general decrease in ribosomal and chaperone proteins in *H*. *megidis* may represent species-specific proteostasis mechanisms. Similarly, organisms accumulate reactive oxygen species (ROS) over time and both species exhibited a gradual increase in proteins which enhance ROS tolerance, such as catalase. The species-specific responses of the proteome in response to storage temperature, and over time, may reflect the phylogenetic distance and/or different ecological strategies.

## Introduction

Entomopathogenic nematodes (Rhabditida; Steinernematidae and Heterorhabditidae) are insect parasites which are of economic importance due to their use as biocontrol agents. The third larval stage, infective juveniles (IJs) leave the natal host and move in the soil to locate a new insect host. Once inside, IJs (at least in *Steinernema*) release a wide array of proteins, which supress the immune system of, and can kill, the insect [1–3]. Both families are associated with mutualistic bacteria, which are released into the haemolymph of the host insect, helping to kill the insect and provide nutrition for nematode development and reproduction [4] and are thus categorised as entomopathogenic nematodes (EPN). Similarities between these two families are due to convergent evolution associated with this lifestyle, rather than common ancestry [5, 6]. Heterorhabditids are closely related to the vertebrate parasites Strongylida [4] and to *Caenorhabditis elegans*, whereas steinernematids are more closely related to Strongyloididae [7]. The IJ stage of parasitic nematodes is analogous to the dauer stage of *C*. *elegans*; both are developmentally arrested, stress resistant stages that disperse to colonise new hosts or food resources, respectively.

The IJs of entomopathogenic nematodes persist in the soil year-round [8], surviving harsh weather conditions. Falling temperatures are an indication of the onset of winter and/or freezing conditions. Exposure to low temperatures improves *Steinernema* and *Heterorhabditis* IJs survival in freezing conditions [9, 10] indicating that there is an acclimatisation mechanism in these IJs. Cold storage also improves the longevity of EPN [11, 12]. IJs are nonfeeding, relying for energy on their internal lipid reserves, which vary in composition amongst EPN species [13]. Dauers and IJs already have lower levels of metabolic activity than other stages of their life cycle [14, 15] and *C*. *elegans* dauers exhibit reduced activity of enzymes involved in glycolytic, gluconeogenic, TCA cycle and oxidative phosphorylation pathways relative to adults [16]. This is referred to as hypometabolism, whereby the organism shuts down all non-essential metabolic activities and redirects their limited resources to essential functions only [17]. In cold conditions, metabolism in EPN IJs slows down further, and their lipids are utilised at a slower rate [18], which is thought to prolong survival. Lack of caloric intake, leading to starvation, is a stressor to which these nonfeeding organisms are inevitably subject, affecting their behaviour and, ultimately, survival [19].

While both steinernematids and heterorhabditids have convergently evolved similar life cycles, there are differences between species in behaviour. For example, of the two species studied here, *Steinernema carpocapsae* IJs are designated as ambushers, waiting on the soil surface for new insect hosts, whereas *Heterorhabditis megidis* IJs are regarded as cruisers, which move within the soil to actively seek out insects [20]. Such differences in the behaviour and in other ecological strategies of the species may be mirrored by molecular differences between them, including their responses to stress. The dauer stage of *C*. *elegans* and IJs of steinernematids and heterorhabditids are often considered non-aging as their lifespan as adults is not significantly affected by the length of time spent developmentally arrested [21]. Nevertheless, these organisms are long-lived when compared to their other developmental stages, and therefore challenges at the molecular level associated with aging may occur. These include protein aggregation and misfolded proteins [21, 22], problems that may be exacerbated in this stage. The overall number of proteins expressed in the dauer/IJ stage of nematodes is lower than in other stages [23] and investigations into *C*. *elegans* confirm that dauers reduce protein synthesis [24] and upregulate the expression of gene families associated with preserving and maintaining cellular components rather than the synthesis of proteins [25, 26]. Much of the research on developmentally arrested juveniles is conducted on dauers of the free-living *C*. *elegans*, and there is relatively little molecular data regarding IJs of parasites [23, 27] including those of EPN [1, 28, 29].

By investigating the molecular mechanisms and consequences of temperature acclimation and time in EPN IJs, insights into survival and the changes induced by low temperature exposure may be gained. Tandem mass spectrometer-based proteomics facilitates the identification and quantification of thousands of proteins in a single run. Such data allows for the comparison of the proteome of EPN IJs after conditioning via gene ontology mapping and functional enrichment analysis. Understanding of how the IJ proteome is affected by temperature and time may elucidate the molecular mechanisms underlying the phenotypic plasticity of EPN IJs [30–32]. This study aims to provide proteomic data for two distantly related species with contrasting ecological strategies, *S*. *carpocapsae* and *H*. *megidis*, stored at 9°C and 20°C for up to 9 weeks.

## Materials and methods

### Nematode culturing and conditioning

*Heterorhabditis megidis* UK211 and *Steinernema carpocapsae* All were used. Nematodes were cultured in last instar *Galleria mellonella* larvae (Mealworm Company, Sheffield, UK) using methods outlined in Woodring and Kaya [33], at 20°C, with an inoculum density of 100 IJs/insect. Cadavers were placed on White traps and monitored daily. After first emergence of IJs, the White trap water was replaced with fresh sterile water. IJs were allowed to emerge into the water for 3–4 days and collected. IJs from successive harvests were pooled, rinsed 3 times by sedimentation and stored at 1000 IJs/ml in sterile tap water in 35 ml aliquots in lidded plastic tubs (9 cm diameter). Tubs were placed at 20°C and 9°C temperature-controlled rooms and sampled at intervals (3 or 6 weeks at 20°C and 3, 6 or 9 weeks at 9°C). In addition, unconditioned IJs (time 0) were also sampled. There were 5 replicate tubs for each storage time and temperature.

### Sample preparation

The contents of a tub were sedimented in a 50 ml Falcon tube in their conditioning temperature. The pelleted IJs in approx. 150 μl were transferred to a 1.5 ml Eppendorf tube and snap frozen in liquid nitrogen.

Each sample was homogenised in lysis buffer, containing 6M urea, 2M thiourea, and a Protease Inhibitor Cocktail (cOmplete, Mini Protease Inhibitor Cocktail, Merck), centrifuged at 10000 x g for 1 minute, and snap frozen. This step was repeated 3 times to ensure complete homogenisation. Protein content was then quantified using Qubit (Invitrogen), following the manufacturer’s instructions. Protein (100 μg) was purified using a 2D Clean Up Kit (GE Healthcare) according to the manufacturer’s instructions. The resulting pellets were stored in the kit’s wash solution at -20°C until the last samples were collected, then all were centrifuged at 13000 x g for 5 minutes and the resulting pellets were resuspended in 50 μl of resuspension buffer (6M urea, 2M thiourea, 0.1M TrisHCl, pH8). A 20 μl aliquot was removed from each sample for reduction, alkylation and digestion. One hundred and five μls of ammonium bicarbonate (50 mM) and 1 μl of dithiothreitol (DTT) were added and samples were incubated at 56°C for 20 minutes. Once cooled, samples were alkylated with 2.7 μl of iodoacetamide (IAA) in dark conditions.

One μl of a 1% (w/v) solution of ProteaseMax (Promega) and 0.5μg/μl trypsin (Promega) were added to the samples and incubated at 37°C for a minimum of 16 hours. Samples were removed from 37°C, centrifuged briefly and acidified with 1 μl of trifluoroacetic acid (TFA) for 5 minutes at room temperature (20–25°C). Samples were centrifuged at 13000 x g for 10 minutes and the supernatant was purified using C18 Spin Columns (Pierce, Thermo Fisher Scientific) following the manufacturer’s instructions and then lyophilised in a Speedyvac concentrator (Thermo Scientific Savant DNA120). Samples were then resuspended in a loading buffer, (2% v/v acetonitrile, 0.05% v/v TFA) and 1 μg was loaded from each of 4 biological replicates per samples were run on a QExactive (Thermo Fisher Scientific) high-resolution accurate mass spectrometer connected to a Dionex Ultimate 3000 (RSLCnano) chromatography system. Peptides were separated over a 2% to 40% gradient of acetonitrile on a Thermo Fisher EASY-Spray, PepMap RSLC C18 column (500mm length, 75mm ID), using a reverse-phase gradient at a flow rate of 250nL min^-1^ over 125 minutes. All data were acquired over 105 minutes with the mass spectrometer operating in automatic data dependent switching mode. A full MS scan at 140,000 resolution and range of 300–1700 m/z was followed by an MS/MS scan, resolution 17,500 and range of 200–2000 m/z, selecting the 15 most intense ions prior to MS/MS.

### Data processing

Protein identification and LFQ normalisation of MS/MS data was performed using Max-Quant v1.6.3.3 (http://www.maxquant.org) following the general procedures and settings outlined in Hubner et al. [34]. The Andromeda search algorithm [35] incorporated in the MaxQuant software was used to correlate MS/MS data for *S*. *carpocapsae* and *H*. *megidis* against the predicted protein data sets derived from the *S*. *carpocapsae* [36] and *H*. *bacteriophora* [37] genomics initiatives, respectively.

Normalised LFQ intensities were used to quantify protein abundances, and the data was filtered to remove contaminants. The LFQ intensities were log_2_ transformed, and each replicate was renamed to their respective groups (3wks9°C for proteins from IJs stored at 9°C for 3 weeks). Only proteins found in 3 replicates of at least 1 group were retained. A data imputation step replaced missing values with values of low abundant proteins chosen randomly from a distribution specified by a downshift of 2 times the mean standard deviation (SD) and a width of 0.3 times the SD.

A principal component analysis (PCA) was initially performed on the normalised intensity values of all replicates. However, a number of outliers were identified, resulting in 3 replicates in each sample in the final datasets for analysis.

An analysis of variance (ANOVA) was performed on all groups using a Benjamini-Hochberg false discovery rate (FDR) of <5% to select proteins for z-score normalisation. These ANOVA significant proteins were used for hierarchical clustering of samples using Euclidean distance and average linkage pre-processed with K means. Gene Ontology (GO) term enrichment was performed in Blast2Go v5.2 using a Fishers exact test (p<0.05) on each cluster relative to all ANOVA significant proteins.

Pairwise Student’s t-tests were performed for all samples relative to the week 0 samples to visualise the effect of time and temperature conditioning on the IJs proteome. Volcano plots were generated in Perseus by plotting negative log p values of the y axis and log_2_fold transformed differences on the x axis for each comparison. Statically significant (SS; p< 0.05) and differentially abundant (DA; fold change of 1.5) proteins were identified as SSDAs and selected for further analysis.

All statistically significant proteins identified in pairwise-t-tests were grouped using Bioedit (v7.0.5.3) and uploaded in FASTA format to STRING: Protein-Protein Interaction Networks Functional Enrichment Analysis v11.0 with the highest confidence setting (0.9) and disconnected nodes were removed, to identify protein-protein interactions which were increasing and decreasing in IJs after storage at 9°C or 20°C over time.

### Bioinformatics

The *H*. *megidis* genome has not been sequenced, and therefore there is a paucity in molecular data available for the species. The genome of the closely related *H*. *bacteriophora* is available [37] and allows for the detection of similar peptides from *H*. *megidis*. The genome of *S*. *carpocapsae* has been recently sequenced [36].

The MS proteomics data and MaxQuant search output files have been deposited to the ProteomeXchange Consortium [38] via the PRIDE partner repository with the dataset identifier PXD027608.

## Results and discussion

In total, 2422 proteins were detected in *S*. *carpocapsae* IJs, of which 2381 remained after filtering and processing, while 1582 proteins were detected in *H*. *megidis* IJs and 653 remained after filtering and processing. The lower number of proteins detected in *H*. *megidis* may be partly a result of using a congeneric (*H*. *bacteriophora)* rather than the subject species as a reference proteome.

**Fig 1:**
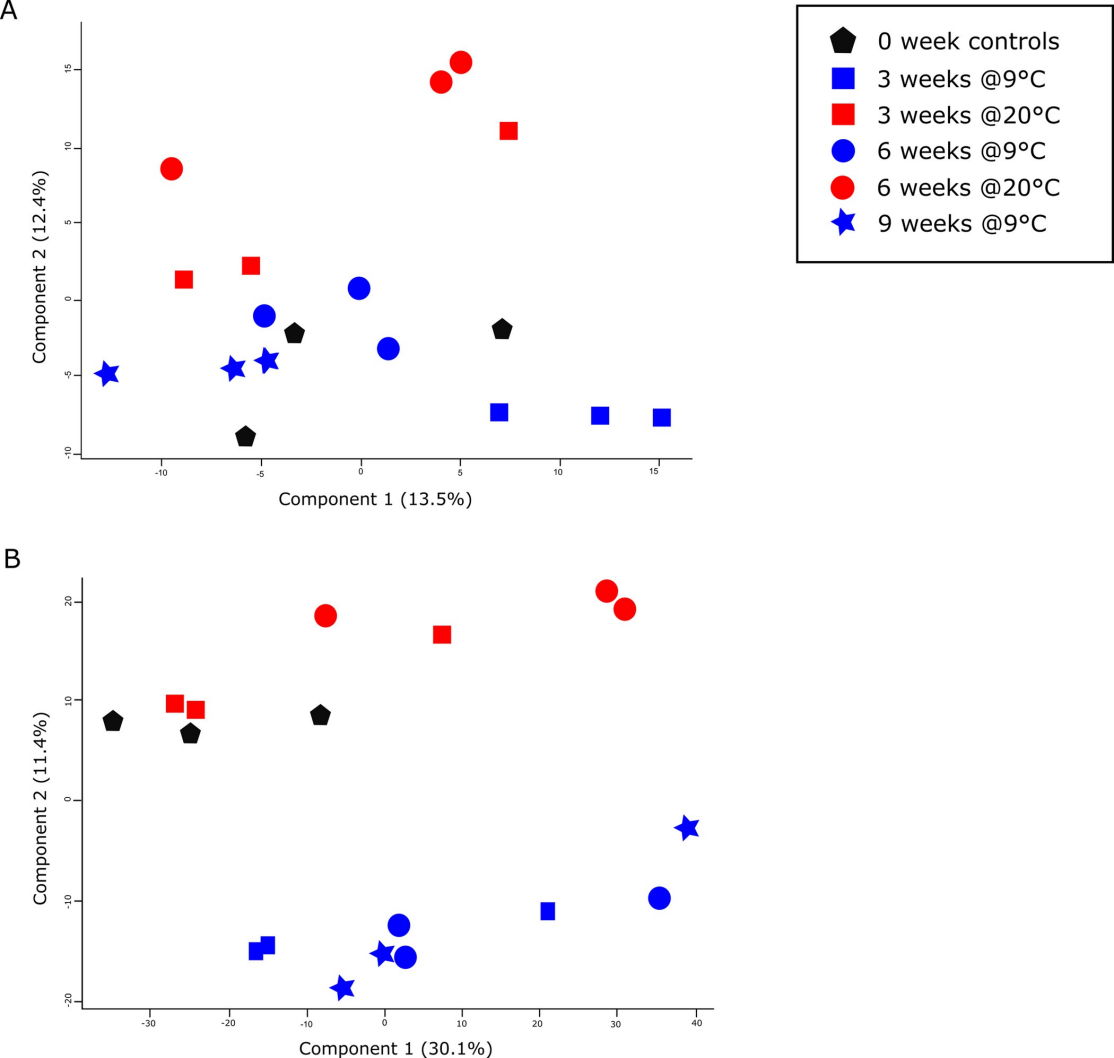
Principal Component Analysis (PCA) of the *H*. *megidis* (A) and *S*. *carpocapsae* (B) proteomes at time 0, or after storage at either 9°C or 20°C for up to 9 weeks. A clear distinction can be seen between IJs stored at 20°C and 9°C.

A PCA for *S*. *carpocapsae* (Fig 1B) showed two distinct groupings of samples, with those stored at 9°C for 3–9 weeks clearly separated from those stored at 20°C or freshly harvested (week 0). There is evidence of progressive change at 20°C from week 0 through week 3 to week 6, while there is less clear differentiation between samples stored at 9°C for different periods. In contrast to *S*. *carpocapsae*, in the PCA for *H*. *megidis* (Fig 1A) there is less of a distinction due to temperature. As in *S*. *carpocapsae*, there is evidence of a progression from 0 to 6 weeks at 20°C, but there is no clear temporal progression for IJs stored at 9°C. Components 1 and 2 for the *S*. *carpocapsae* PCA accounts for 41.5% of the data’s variability. Components 1 and 2 for the *H*. *megidis* PCA account for 25.9% of the data’s variability.

**Fig 2:**
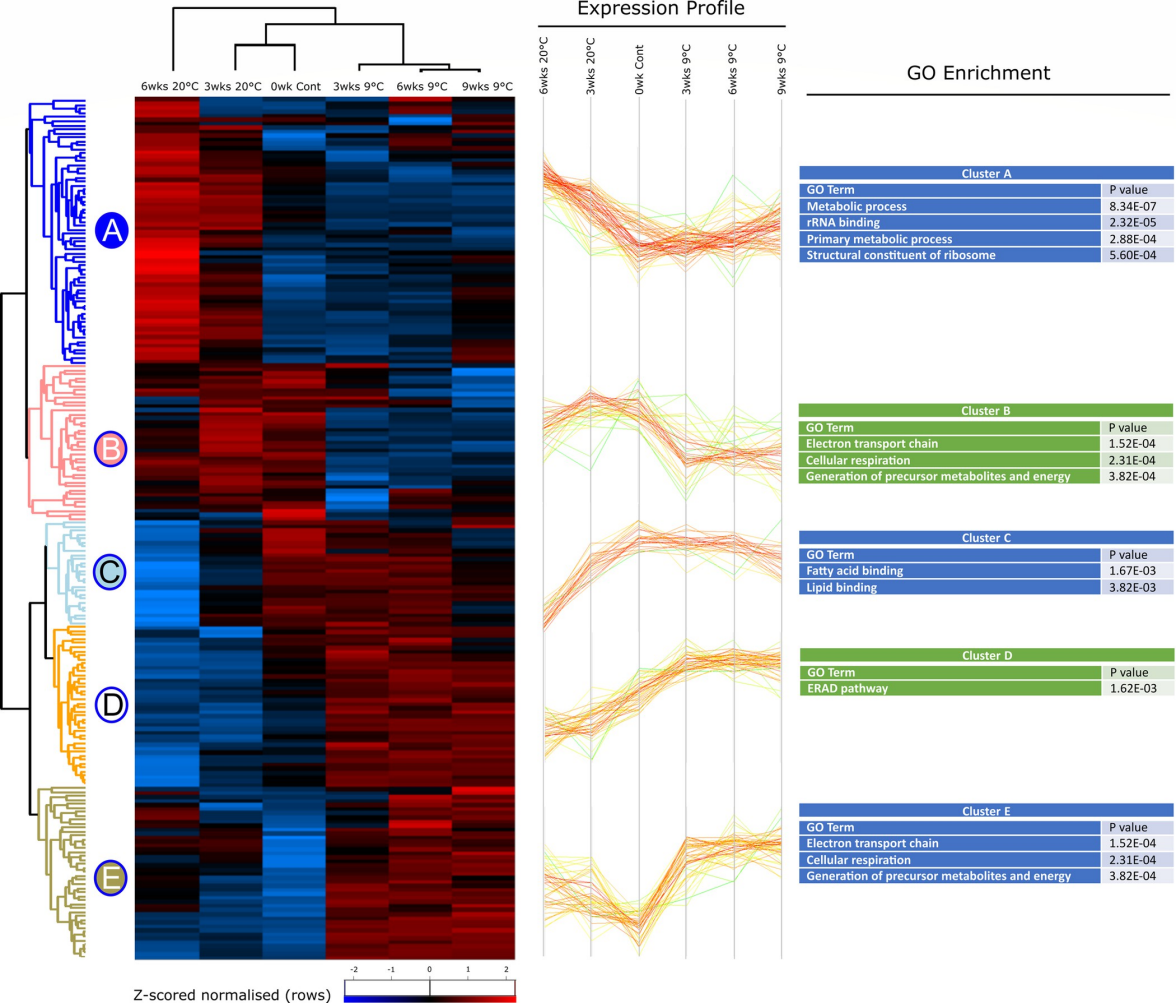
Heat map of *S*. *carpocapsae* statistically significant proteins. Two-way unsupervised hierarchical clustering of the median Z-score normalised label-free quantification (LFQ) intensity values of all statistically significant proteins (n = 214) for freshly emerged IJs, IJs stored at 9°C for 3, 6 and 9 weeks or IJs stored at 20°C for 3 and 6 weeks. Hierarchical clustering resolved 5 distinct clusters. Differences in protein abundance are indicated by colour changes from low (blue) to high (red) protein abundance representative of changes in Z-score normalised log_2_-fold transformed LFQ intensity values. Selected GO terms enriched in each cluster are displayed (right), along with the P value for that category.

**Fig 3:**
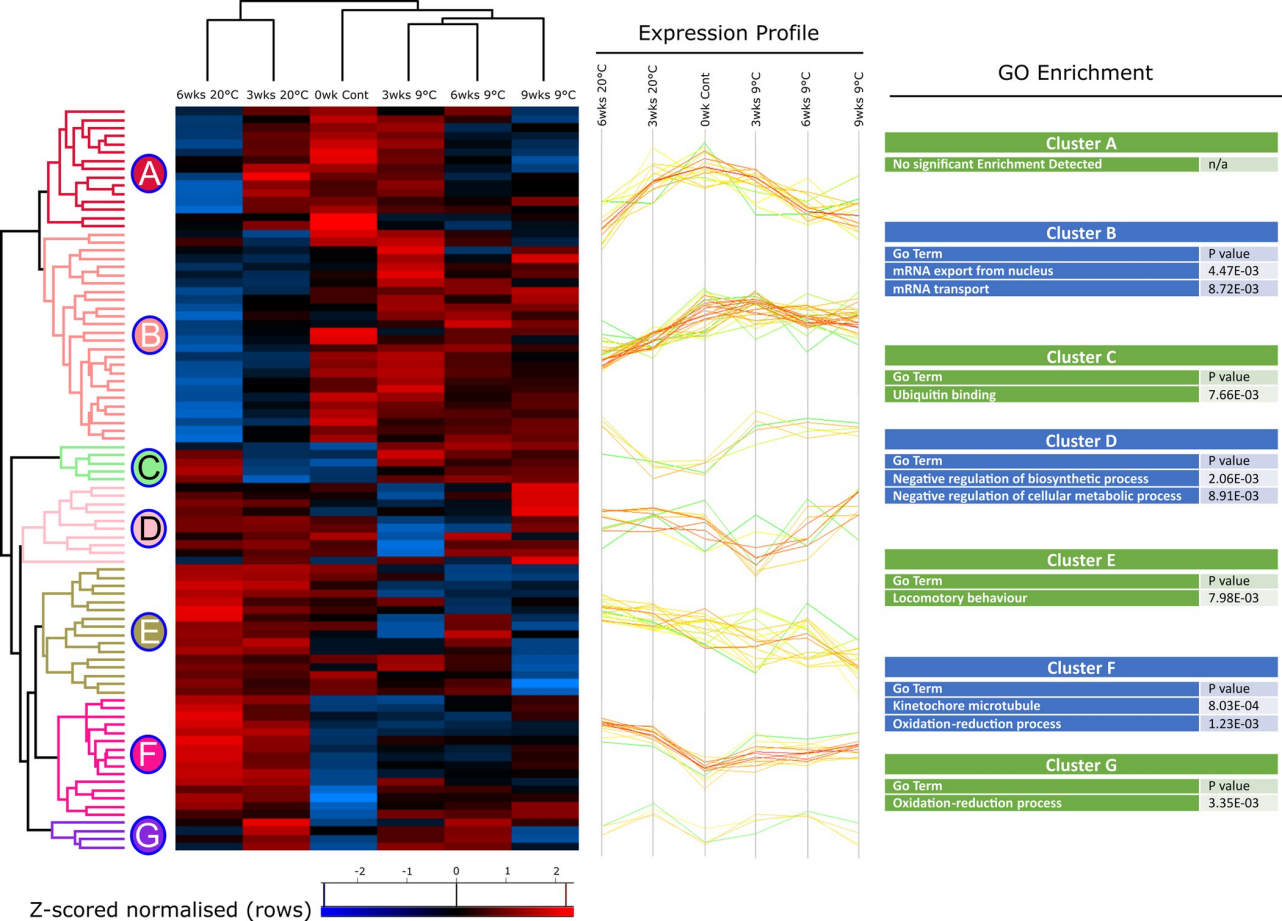
Heat map of *H*. *megidis* statistically significant proteins. Two-way unsupervised hierarchical clustering of the median Z-score normalised label-free quantification (LFQ) intensity values of all statistically significant proteins (n = 91) for freshly emerged IJs, IJs stored at 9°C for 3, d 9 weeks or IJs stored at 20°C for 3 and 6 weeks. Hierarchical clustering resolved 5 distinct clusters. Differences in protein abundance are indicated by colour changes from low (blue) to high (red) protein abundance representative of changes in Z-score normalised log_2_-fold transformed LFQ intensity values. Selected GO terms enriched in each cluster are displayed (right), along with the P value for that category.

Temporal changes in proteins are shown in more detail as heatmaps (Figs 2 and 3), revealing 5 clusters of proteins in *S*. *carpocapsae*, and 7 clusters in *H*. *megidis*. The clusters group proteins which are detected with similar abundance-profiles in each group. Distinct time and temperature-dependent responses are seen in *S*. *carpocapsae;* for example, cluster A increases over time at 20°C while remaining rather stable at 9°C, while in contrast clusters C and D decrease over time at 20°C (Fig 2). Patterns are more complex in *H*. *megidis* (Fig 3).

Pairwise comparisons to time 0 allowed the numbers of proteins changed in each stored sample (SSDA) to be quantified (Table 1). There were 724 SSDA proteins for *S*. *carpocapsae* and 175 for *H*. *megidis*, representing 30.4 and 26.8%, respectively, of the (filtered) proteome for each species. The identity and fold change of a selection of these proteins is given in S1 and S2 Tables. In *S*. *carpocapsae*, there was a tendency for twice as many proteins to be increased in abundance as were decreased in abundance in each storage treatment, while in *H*. *megidis* the numbers increased and decreased in abundance tended to be more equal (Table 1).

**Table 1:** Number of significantly significant (P < 0.05) differentially abundant (SSDA) proteins showing increased or decreased activity (fold change +/- 1.5-fold) relative to time 0 following storage at 9°C or 20°C of infective juveniles of *S*. *carpocapsae* or *H*. *megidis*.

Storage temp (°C)	Storage duration (wks)	*S*. *carpocapsae*		*H*. *megidis*	
		Up	Down	Up	Down
20	3	80	46	27	22
	6	229	105	57	49
9	3	104	51	25	24
	6	225	85	21	13
	9	167	80	21	48

In general, SSDA proteins demonstrated a greater fold change in *S*. *carpocapsae* than in *H*. *megidis*. The greatest change in *S*. *carpocapsae* was for chaperone proteins which showed up to 90-fold increase after conditioning at 9°C for 9 weeks (S1 Table), while all chaperone proteins detected in *H*. *megidis* were decreasing in abundance (S2 Table). In *H*. *megidis*, the greatest change in any single protein was that of UDP-glucoronosyl and UDP-glucosyl transferase which increased ~20 fold at 20°C, and ~7 times at 9°C (S2 Table).

### Translation

**Fig 4:**
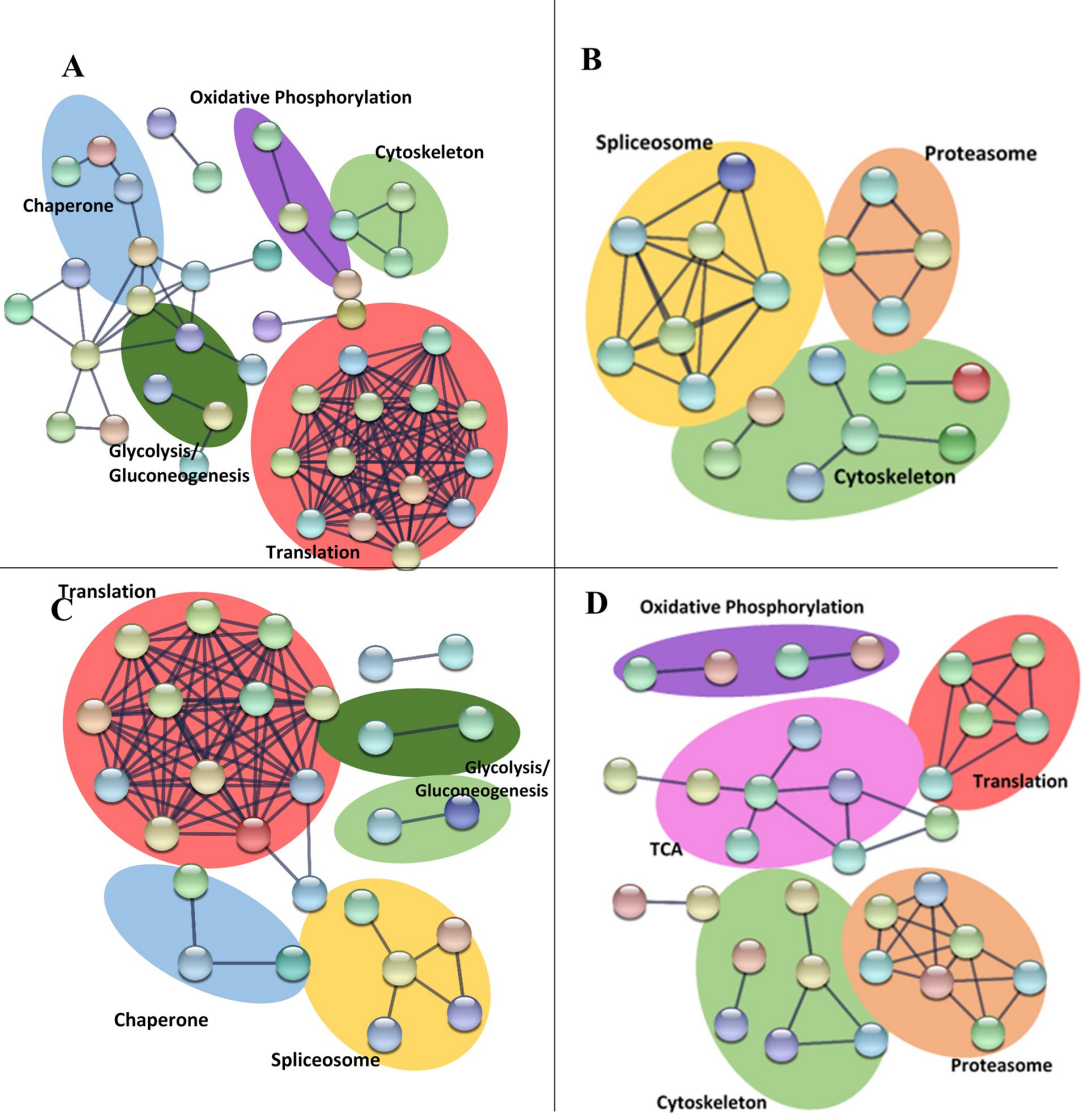
String interactions of *H*. *megidis* proteins which are decreased (left) and increased (right) in abundance after storage at 9°C (top) and 20°C (bottom) for 6 weeks.

**Fig 5:**
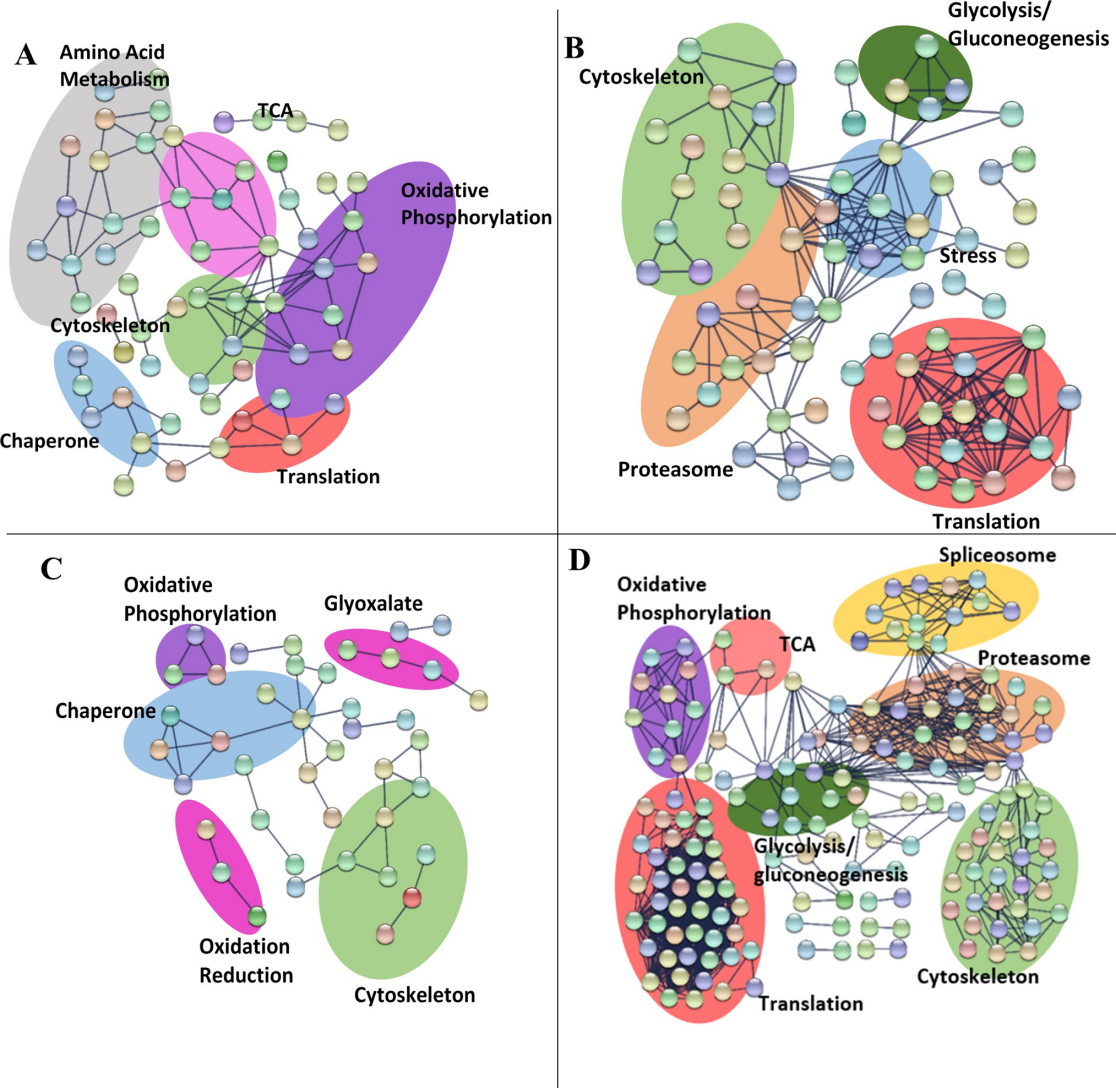
String interactions of *S*. *carpocapsae* proteins which are decreased (left) or increased (right) in abundance after storage at 9°C (top) and 20°C (bottom) for 6 weeks.

The most prominent difference between the two species is in the response of proteins related to translation, which were decreased in *H*. *megidis* after storage in both 9°C and 20°C (Fig 4) and were generally increased in *S*. *carpocapsae* (Fig 5; S1 Table). All ribosomal proteins decreased in abundance over time in *H*. *megidis* IJs (S2 Table) regardless of storage temperature. Protein production is energetically expensive, costing up to 75% of the cell’s energy [39]. As metabolic activity is reduced, reduction of energetically expensive processes would be advantageous. Walther et al. [22] reported extensive proteome remodelling in aging *C*. *elegans* worms, with reduced ribosomes and increased proteasome complexes. Most proteasome related proteins detected were increased at both temperatures in both species. Reduction of translation and mRNA production has shown to extend the lifespan of *C*. *elegans* [24, 40]. *S*. *carpocapsae* IJs did not exhibit a general decrease in ribosomal proteins over time, and translation related proteins were generally increased in abundance, regardless of storage temperature (S1 Table; Fig 5).

### Chaperones

Most chaperone proteins that were detected were increased in abundance in *S*. *carpocapsae* IJs, and to a greater extent after storage at 9°C (S1 Table). These include chaperonins, prefoldins, heat shock proteins and late embryogenesis (LEA) proteins. One chaperonin was detected in *S*. *carpocapsae*, increasing in abundance at 20°C, and in *H*. *megidis*, decreasing at 9°C. Chaperonins are ATP-dependent chaperones which assist in the folding of nascent polypeptides [41]. Various heat shock proteins (HSPs) were detected in *S*. *carpocapsae* (S1 Table) and generally increased in abundance over time, and to a greater extent at 9°C. HSPs are molecular chaperones which aid folding of proteins, prevent stress induced aggregation or misfolding of proteins, and can revert misfolded proteins to their native conformation [42]. HSPs, despite their name, are protective against a wide array of stresses and can also have roles in maintaining cellular components such as the cytoskeleton [43]. Small heat shock proteins are typified by a conserved α-crystallin C terminal domain [42] and are involved in the prevention of toxic aggregates of protein [44], and have higher binding affinities than the larger, classical HSPs [42]. Prefoldins are molecular chaperones which detect, bind to and deliver unfolded proteins, especially actin, to cystolic chaperonins [45]. Prefoldin proteins were amongst the few chaperone proteins decreased in abundance in *S*. *carpocapsae* IJs, at both 9°C and 20°C.

Many late embryogenesis abundant (LEA) proteins were detected in *S*. *carpocapsae* IJs, and most were temperature specific: increasing by up to ~90 times abundance after storage at 9°C, but mostly decreased by ~2–4 times if stored at 20°C (S1 Table). LEA proteins were first discovered in the seeds of plants [46, 47] and confer desiccation tolerance to the seeds. Since their initial discovery, these proteins have been found in a variety of nematodes such as *C*. *elegans* [48], *Steinernema feltiae* [49] and *Aphelenchus avenae* [50]. LEA proteins are atypical molecular chaperones which can protect the structure of proteins [51], if present before the exposure to stress. Unlike heat shock proteins, LEA proteins alone cannot protect proteins from heat stress and cannot revert misfolded proteins back to their native state [52, 53]. LEA proteins enhance the organism’s survival in response to cold and freezing conditions [54, 55], oxidative stress [56] and salt stress [57]. LEA proteins, in conjunction with trehalose, facilitate the formation of “glass” [50], which is protective against desiccation [48] and freezing [58]. Trehalose-6-phosphate synthase, which was increased at 6 weeks in *S*. *carpocapsae* regardless of storage temperature (S1 Table), may be involved in this bioglass formation or could be a response to starvation.

All chaperone proteins detected as SSDAs in *H*. *megidis* were decreased in abundance, regardless of storage temperature (Fig 4; S2 Table). With the sharp decrease in translation, chaperone proteins may be redundant to *H*. *megidis* IJs without an immediate stressor such as heat or desiccation triggering their expression. Decreased translation would also reduce the level of misfolded proteins and toxic aggregates, which may reduce the need for protein chaperone activities.

### Metabolism

Proteins associated with gluconeogenesis are amongst the few groups of metabolism related proteins which were increased in abundance in *S*. *carpocapsae* IJs (Fig 5; S1 Table) and their increase may be related to starvation of the worm. Gluconeogenesis is responsible for the generation of monosaccharides used to generate energy in subsequent metabolic processes. Sugars formed in gluconeogenesis are converted to pyruvate during glycolysis, which is then converted to acetyl-CoA by pyruvate dehydrogenase. Pyruvate formed by glycolysis is transported to mitochondria to be converted to acetyl-CoA in the citric acid cycle, an aerobic process which utilises acetyl-CoA to reduce NAD+ to NADH, producing carbon dioxide as a by-product. The NADH is then used in oxidative phosphorylation. While glucose is the classic sugar transport unit formed in this pathway, nematodes and insects also form trehalose, a disaccharide composed of 2 glucose molecules. This sugar is functionally similar but also plays a crucial role in nematodes’ resistance against desiccation, freezing [59], and other stresses [60]. This sugar, along with LEA proteins facilitates the formation of bioglass, which enhances nematodes’ freezing tolerance [50].

Few glycolysis-related proteins were detected as SSDAs in *H*. *megidis* and these were generally decreasing in abundance at both temperatures (Fig 4; S2 Table). Most glycolysis proteins detected in *S*. *carpocapsae* did not change significantly over time at either temperature (Fig 5; S1 Table). The glyoxylate pathway is an alternative pathway to the TCA cycle, which is not normally found in metazoans, but is present in nematode dauers [61]. Isocitrate lyase and malate synthase catalyse the conversion of coenzyme A to succinate and malate [62], which may then be processed by succinate dehydrogenase to form malate, which can then be processed by malate dehydrogenase. This pathway allows for the generation of glucose molecules from the β-oxidation of fatty acids. There is significant overlap between the enzymes in these two metabolic pathways, and most enzymes were increased in abundance during storage at 20°C and decreased at 9°C in *S*. *carpocapsae* IJs (S1 Table). All SSDA proteins detected as part of both the citric acid cycle and the glyoxylate pathway were decreased in abundance regardless of storage temperature in *H*. *megidis* IJs (S2 Table).

Few oxidative phosphorylation enzymes were detected in *H*. *megidis* (S2 Table), but those that were detected increased in abundance at 20°C and decreased in abundance at 9°C (Fig 4; S2 Table). The exception to this was NADH-ubiquinone oxidoreductase ASHI subunit, which was increased by ~11 times at both temperatures. Proteins related to oxidative phosphorylation were abundant in a temperature specific manner in *S*. *carpocapsae* IJs, increasing at 20°C and decreased in abundance at 9°C (Fig 5; S1 Table).

Many fatty acid- and retinol-binding proteins (FARs) and nematode polyprotein allergens/antigens (NPAs) were detected in *S*. *carpocapsae* (S1 Table), most decreasing in abundance at both storage temperatures. FARs are a diverse family of proteins that are expanded in the *S*. *carpocapsae* genome [63]. NPAs are spliced to form many copies of nematode FARs [64]. Nematode FARs have structures similar to FARs found in other animals but have structures unique to nematodes, and therefore probably have nematode specific functions. FAR proteins may transport and store small quantities of lipids [65].

In general, *S*. *carpocapsae* proteins associated with metabolism decreased at 9°C and increased at 20°C (S1 Table). Proteins related to both gluconeogenesis and glycolysis were increased in abundance in *S*. *carpocapsae* IJs stored at 9°C, whereas proteins related to intermediary metabolism and oxidative phosphorylation were decreased to a greater extent at 9°C (Fig 5). This may be due to IJs being more active at higher temperatures and requiring more energy [18] and may be partially responsible for the IJs’ enhanced longevity at low temperatures [11, 12].

### Cytoskeleton

Cytoskeletal proteins such as actin, myosin, collagen, and tubulin were amongst the highest abundance proteins detected in both species by raw LFQ intensity. Proteins associated with the cytoskeleton were generally decreased in abundance in both species over time (Figs 4 and 5; S1 and S2 Tables). EPN IJs tend to become less active over time [19], and therefore proteins associated with locomotion may be degraded. Collagen remodelling is also reported to be associated with lifespan-lengthening in *C*. *elegans*, regulated by the stress pathway SKN-1 [66]. Collagen was detected as decreased in abundance in *S*. *carpocapsae* IJs stored at 20°C, however it was increased in those stored at 9°C (S1 Table).

### Stress/Detoxification proteins

String analysis identified a network of stress proteins which increased in abundance in *S*. *carpocapsae* at 9°C (Fig 5), although there was a tendency for stress/detoxification proteins to also increase in abundance at 20°C. Many stress proteins detected as SSDAs in this analysis in both species such as short chain dehydrogenases, thioredoxins, GSTs, catalase, oxidoreductases, aldehyde dehydrogenases (S1 and S2 Tables), are known to be regulated by SKN-1 [67], the pathway implicated in collagen remodelling mentioned above [66]. Proteins associated with the cell’s response to reactive oxygen species, such as catalase in *H*. *megidis*, and both catalase and copper oxide dismutase in *S*. *carpocapsae*, were increased to a greater extent at 9°C than at 20°C. Thioredoxin, an antioxidant, was decreased in abundance at both temperatures at all timepoints in both *H*. *megidis* and *S*. *carpocapsae*. Other stress proteins such as aldehyde dehydrogenase, which is involved in the stress response against by-products of anaerobic fermentation were increased to a greater extent at 20°C than at 9°C in *S*. *carpocapsae* (S1 Table).

Xenobiotic detoxification is generally divided into three distinct stages. Short chain dehydrogenases and reductases render xenobiotics less stable and represent phase I. Glutathione S transferase is involved in stage II, and transfers glutathione (an antioxidant) onto xenobiotics, increasing their solubility and facilitating its breakdown. Glutathione can also reduce free radicals generated during the stress response. UDP-glucoronosyl and UDP-glucosyl transferase increased in abundance in both *H*. *megidis* and *S*. *carpocapsae* IJs, at most timepoints and both temperatures (S1 and S2 Tables). UDP-glucoronosyl transferases add glucuronic acid to a xenobiotic, which may render it harmless, or aid in its excretion [68]. Glucosyltransferases may also be involved in the synthesis of trehalose [69], a sugar which enables survival in harsh conditions [60].

Fewer stress-related proteins were detected in *H*. *megidis*, and many of them were decreased in abundance after conditioning at both temperatures, except for catalase, which increased after storage at 9°C (S2 Table). Amongst these proteins, one of the few SSDA proteins that was constitutively increased in abundance over time was an autophagy related protein, and it increased to a greater extent at 9°C than at 20°C (S2 Table). Selective autophagy has been shown to improve *C*. *elegans* lifespan at low temperatures [70] and may have a similar role in *H*. *megidis* IJs.

## Conclusion

When infective juveniles of *H*. *megidis* UK211 and *S*. *carpocapsae* All were conditioned at 9°C or 20°C, the proteome of these two species changed in radically different manners. The change in the *H*. *megidis* proteome was characterised by a decrease in proteins associated with metabolism and protein synthesis, while the change in *S*. *carpocapsae* was characterised by increases in proteins associated with protein chaperoning activities and responses to stress which increased over time, and to a greater extent at 9°C. The difference in proteostasis may be due to several factors.

Firstly, since the two species are not closely related, their strategies may be legacy of their ancestry. Secondly, it may relate to differences in the behavioural (foraging) strategies of the two species. *H*. *megidis* IJs are defined as “cruisers”, IJs which actively move through soil to find their host, whereas *S*. *carpocapsae* IJs are defined as “ambushers”, IJs which wait until a potential host comes near enough to infect [20] although this is likely an oversimplification of their complex behaviours [32]. As protein production is energetically expensive, requiring up to 75% of the cell’s energy [39], a reduction in protein synthesis may free up energy for locomotion in *H*. *megidis* and may enable the IJ to avoid proteotoxic stress such as misfolded or aggregating proteins [22]. All chaperone proteins were decreased in abundance in *H*. *megidis*, regardless of storage temperature. *S*. *carpocapsae*, described as more of a sedentary ambusher, may not require this extra energy. Without reducing protein synthesis, *S*. *carpocapsae* IJs may be affected by proteotoxic stress. This may be why chaperone proteins were increased to such an extent in *S*. *carpocapsae*, regardless of storage temperature. Thirdly, the differences between the two species may relate to differences in broader ecological strategies. *H*. *megidis* IJs disperse widely and all are hermaphroditic, characteristics of r-strategists or a colonising species, and so perhaps this species spends relatively little time as IJs in soil [71]. *S*. *carpocapsae* IJs are relatively long-lived compared to *H*. *megidis* and would thus be better suited to persist in soil during periods when hosts are unavailable such as winter. Storage temperature had a clear effect on the *S*. *carpocapsae* proteome, which showed a much greater increase in chaperone abundance at 9°C, especially in LEA proteins. As these IJs are present in the soil year-round, exposure to low temperatures may indicate the onset of freezing conditions, and the IJ’s proteome may adapt to this. LEA proteins, which are important for freezing resistance in nematodes were increased in a temperature specific manner.

Oxidative phosphorylation produces the most energy of all metabolic processes, but it also produces detrimental reactive oxygen species. Free radicals damage cells and contribute towards aging. *Steinernema* and *Heterorhabditis* IJs generally live longer at 9°C than at 20°C, which is generally attributed to their metabolism slowing down at lower temperatures [18, 72, 73]. Catalase, the stress enzyme which breaks down hydrogen peroxide, and copper oxide dismutase were both increased in abundance after storage at 9°C in *S*. *carpocapsae*. IJs may enhance their survival in colder conditions by reducing metabolic activities which produce these free radicals and increasing their stress response against them. Further studies on a broader range of species (including species of *Steinernema* that adopt cruise-foraging strategies) would help elucidate to what extent the patterns detected in the two species studied here reflect their ancestry and/or their ecological strategies.

## Supporting information

S1 Table Relative abundances of *S*. *carpocapsae* proteins. Log_2_fold transformed abundances of proteins identified as statistically significant (P<0.05 in student t-tests) relative to week 0 in *S*. *carpocapsae* IJs after conditioning.
(XLSM)

S2 Table Relative abundances of *H*. *megidis* proteins. Log_2_fold transformed abundances of proteins identified as statistically significant (P<0.05 in student t-tests) relative to week 0 in *H*. *megidis* IJs after conditioning.
(XLSM)
